# Alexander Mactier Pirrie

**Published:** 1908-01

**Authors:** 


### Alexander Mactier Pirrie.

A CAREER of great promise has been cut short by the untimely death of Mr. A. Mactier Pirrie, who was a martyr to science. The son of the late Mr. Alexander Pirrie, C.E., he was born on October 2, 1882. He obtained his B.Sc., with honors in anthropology at Edinburgh University in 1904, and qualified as M.B., Ch.B. in 1906. He obtained the Carnegie Research Fellowship in anthropology and was appointed anthropologist to the Wellcome Research Laboratories at the Gordon Memorial College, Khartoum. He went out to the Soudan in the autumn of 1906.

Under the direction of Dr. Andrew Balfour, the Director of the Laboratories, Dr. Pirrie made his first expedition up the Nile to the southern limits of the Soudan and penetrated to remote parts of the Bahr-el-Ghazal. His second expedition took him to the borders of Abyssinia. On both occasions he passed through some of the most pestilential regions of Africa in connection with certain anthropological and physiological researches, appertaining to tropical diseases, upon which the laboratories are engaged.

Unfortunately he contracted tropical fever (kala-azar) and was so prostrated as to be compelled to return to England, leaving Khartoum on June 17, 1907. He rallied from the effects of the fever from time to time, but was compelled to enter Chalmers' Hospital, Edinburgh, in October. His death took place November 12, 1907.

He was interred at the Dean Cemetery, Edinburgh, November 15. The Gordon Memorial College, Khartoum, Sir William Turner, Principal and Vice-chancellor of the University, Mr. Wellcome and others were represented, and sent wreaths. A resolution of sympathy has been conveyed to the relatives from the trustees of the Gordon Memorial College, and expressions of sympathy have been received from the Liverpool School of Tropical Medicine, and other institutions and societies.

It is of interest to note that the first case of kala-azar found in Africa, except a case in Tunis, referred to by Laveran, was reported by Dr. Sheffield Neave, pathologist to the Wellcome Research Laboratories, Khartoum. Dr. Neave found the Leishman-Donovan body, the parasite of kala-azar, in the splenic blood of a patient in the Omdurman Civil Hospital. The discovery is noted by the Director in the Second Report of the Laboratories.

Dr. Pirrie presented a paper on his African expeditions at the last meeting of the British Association for the Advancement of Science, but was prevented from being present on account of his illness. He brought back a most valuable collection of objects of scientific interest. At intervals during his illness he was engaged on his report to the Carnegie Institute and the Wellcome Research Laboratories, Khartoum, for which institutions he acted jointly in the important work he carried out in the Soudan.

---

Whitelaw Reid, American Ambassador to the Court of St. James's and Chancellor of the University of the State of New York, who on December 20, 1907, returned to America for the holidays, presided December 21st, at the annual meeting of the State Board of Regents at Albany. These appointments on college and academic councils were announced:

Convocation—Edward L. Stevens, New York; college, President Langdon C. Stewardson of Hobart College; academic, E. R. Whitney, Binghamton; library, Frank P. Hill, Brooklyn (reappointed); medical, Dr. Egbert Le Fevre, New York.

Advisory Council of Nurse Training Schools—Annie W. Goodrich, New York; Mrs. C. N. Simpson, Albany; Miss M. L. Jones, Rochester; Ida M. Root, Gloversville; Dr. William L. Russell, Poughkeepsie.

The Regents also filled vacancies on state professional boards as follows:

State Board of Nurse Examiners—Mary E. May, of the Willard State Hospital.

State Board of Examiners of Certified Public Accountants—Henry A. Niles and Duncan MacInnes, of New York City; Thomas B. Dixey. Albany.

The Regents also approved the establishment of reciprocal relations in medical licensure between the states of New York and Vermont.

---

Dr. Eugene H. Porter, state commissioner of health, has taken another important step in the warfare against tuberculosis. Late last month, that is to say, December 23, 1907, he appointed an advisory board, composed of experts on tuberculosis, "to give to the state department of health such advice and suggestions as in their wisdom and expert knowledge, they may deem proper and expedient." All those appointed have signified their willingness to serve. They are:

Edward B. Baldwin, Saranac; George W. Goler, Rochester; Willis G. Macdonald, Albany; Veranus M. Moore, Ithaca; John

H. Pryor, Buffalo; William H. Watson, Utica; Thomas Darlington, Alfred Meyer, Livingston Farrand and Homer Folks of New York. Dr. Porter stated that Governor Hughes has approved the course he has taken and that the legislature will be asked for an appropriation of $50,000 to carry on the proposed work.

---

THE State of Illinois has amended its practice act, authorising the state board of health to establish a standard of preliminary education and to require satisfactory proof of the enforcement of this standard by medical colleges. It is left to the board to determine as to what constitutes satisfactory proof; but it is provided that examinations of applicants for admission conducted by the faculty of a medical college shall not be recognised. The diploma of an approved four-year high school or its equivalent, or the certificate of a state superintendent of public instruction shall be accepted as satisfactory evidence of preliminary qualifications. This confers upon the board appropriate powers and fixes the responsibility as to preliminaries where it belongs. This legislation is in the line of progress.

---

CHAMPE S. ANDREWS, Esq., Counsel for the Medical Society of the County of New York, in his annual report recently made to the society (*Medical Record*, December 14, 1907, p. 1006) states that the total number of persons prosecuted since the last annual report was 80 under the old law, and 700 under the new law; the total number convicted under the old law was 47, under the new law 439; cases disposed of otherwise than by conviction, 9 under the old law and 180 under the new law. There were 20 cases still pending, and four persons were imprisoned. The aggregate fines imposed under the old law amounted to $4,675; under the new law, $35,062.95.

---

APOLLINARIS, the Queen of table waters, has never been surpassed as a beverage for sick or well persons. No well appointed dinner function is complete without it. It has never ceased to be a favorite with people of refinement since it was first bottled for general use. It is the water above all others that should be prescribed by physicians to patients with delicate stomachs who need a sparkling non-vinous drink. Apenta is another water supplied by the Apollinaris Company, which is invaluable when a mild laxative is needful, it being the best natural aperient water bottled in Hungary.

---

SUPERINTENDENT EMERSON, in his annual report as superintendent of education, calls attention to the lack of regular medical inspection in the schools of Buffalo. If it is admitted that schools are natural centers of infection, then there should be adequate medical inspection. Buffalo should no longer shirk its duty in this matter. It is as important to inspect the schools as the tenement houses; it is more important than to provide ward physicians to attend the sick; just as it is more important to attempt to prevent disease than to assume to cure it.

---

MOET & CHANDON White Seal Champagne, vintage of 1900, is a wine that should claim first attention from those who enjoy and prefer the best at every dinner where champagne is served; and especially should it be served at social functions during the holiday season. But of most interest to physicians is the fact that, owing to its purity and its delicacy of flavor, it is the wine, *par excellence,* for the sickroom whenever a sparkling, fruity champagne is needed.

---

THE French army medical corps, according to the *Tribune,* intends to develop the use of dogs in connection with ambulance work. This step is taken in consequence of the success of some trials carried on at the autumn manœuvres. An intelligent collie named Nelly, which, despite its English name, is German born and bred, specially distinguished herself after one sham fight by guiding the ambulance men to a hundred "wounded" men hidden in a wood. This dog was trained in less than a month.

---

AFTER being warned so much and so long as to the danger of infection from soiled banknotes, it comes with something of a jolt to hear from Dr. Doty, as published elsewhere in this issue, that there is "nothing in it," that dirty money does not spread disease, and that we need not be afraid of handling it. Well, we are glad to hear it; it will take away some of the scare we have always felt about filthy leucre.

---

CALOX, the oxygen tooth powder, is recommended by physicians because it is constructed on sound chemical principles, combining lime and oxygen in such a manner as to destroy pyogenic surfaces without doing harm to the adjacent tissues. It is a dentifrice which should be found among the toilet articles of every refined man and woman.

